# High fluoroquinolone MIC is associated with fluoroquinolone treatment failure in urinary tract infections caused by fluoroquinolone susceptible *Escherichia coli*

**DOI:** 10.1186/s12941-017-0202-4

**Published:** 2017-04-08

**Authors:** Pinyo Rattanaumpawan, Irving Nachamkin, Warren B. Bilker, Jason A. Roy, Joshua P. Metlay, Theoklis E. Zaoutis, Ebbing Lautenbach, on behalf of the CDC prevention epicenters program

**Affiliations:** 1 Center for Clinical Epidemiology and Biostatistics, Perelman School of Medicine, University of Pennsylvania, Philadelphia, PA USA; 2 Division of Infectious Diseases and Tropical Medicine, Department of Medicine, Faculty of Medicine Siriraj Hospital, Mahidol University, Bangkok, Thailand; 3 Department of Pathology and Laboratory Medicine, Perelman School of Medicine, University of Pennsylvania, Philadelphia, PA USA; 4 Department of Biostatistics, Epidemiology, and Informatics, Perelman School of Medicine, University of Pennsylvania, Philadelphia, PA USA; 5 Division of General Internal Medicine, Massachusetts General Hospital, Boston, MA USA; 6 Division of Infectious Diseases, Department of Medicine, Perelman School of Medicine, University of Pennsylvania, Philadelphia, PA USA

**Keywords:** *Escherichia coli*, Fluoroquinolone resistance, Urinary tract infection

## Abstract

**Background:**

Suboptimal clinical response to fluoroquinolone (FQ) therapy has been clearly documented in patients with Salmonella typhi infection with reduced FQ susceptibility. However, the clinical impact of reduced FQ susceptibility on other infections including *E. coli* urinary tract infections (UTIs) has never been evaluated.

**Methods:**

We conducted a retrospective cohort study of female patients with fluoroquinolone susceptible *E. coli* (FQSEC) UTIs who received FQ therapy at outpatient services within University of Pennsylvania Health System, Philadelphia. Exposed patients were those with high MIC-FQSEC UTIs (the levofloxacin MIC > 0.12 but ≤ 2 mg/L) while unexposed patients were those with low MIC-FQSEC UTIs (the levofloxacin MIC ≤ 0.12 mg/L). The primary treatment outcome was treatment failure within 10 weeks after initiation of FQ therapy.

**Results:**

From May 2008 to April 2011, we enrolled 29 exposed patients and 246 unexposed patients. Two patients in each group experienced treatment failure; exposed vs. unexposed (6.9 vs. 0.8%; *p* = 0.06). Risk difference and risk ratio (RR) for treatment failure were 0.06 [95% CI −0.03–0.15; exact*-p* = 0.06] and 8.48 [95% CI 1.24–57.97; exact*-p* = 0.06], respectively. After adjusting for underlying cerebrovascular disease, the RR was 7.12 (95% CI 1.20–42.10; MH-*p* = 0.04).

**Conclusion:**

Our study demonstrated the negative impact of reduced FQ susceptibility on the treatment response to FQ therapy in FQSEC UTIs. This negative impact may be more intensified in other serious infections. Future studies in other clinical situations should be conducted to fill the gap of knowledge.

## Background

Fluoroquinolone (FQ) susceptibility is traditionally reported as susceptible, intermediate, or resistant [1]. Some investigators further categorized the fluoroquinolone susceptible *E. coli* (FQSEC) isolates into two additional subgroups, based on the MIC cutoff value; (1) Fully susceptible strain or low MIC-FQSEC group (the MIC level against levofloxacin ≤ 0.12 mcg/mL); and (2) Reduced susceptible strain or High-MIC FQSEC group (levofloxacin MIC > 0.12 but ≤ 2 mg/L) [2, 3]. These reduced susceptible strains potentially result in development of full resistance to FQ and may lead to delayed response to FQ therapy [4].

Negative impact of high MIC-FQ susceptibility on treatment outcomes has been previously documented in several studies [5, 6]. The previous study from Vietnam revealed that high MIC-FQ susceptibility was associated with treatment failure in patients with enteric fever who received ofloxacin therapy [6]. However, another recent study from Vietnam did not find any association between poorer outcome and reduced FQ susceptibility among pediatric patients with Shigella infections [7]. Furthermore, the impact of high MIC-FQ susceptibility on clinical outcomes among patients with infection caused by *E. coli* has never been thoroughly investigated.

Given these considerations, we conducted a retrospective cohort study aiming to determine the clinical impact of high MIC-FQ susceptibility on FQ treatment response among female patients with fluoroquinolone susceptible *E. coli* urinary tract infections (FQSEC UTIs) in ambulatory settings.

## Methods

### Study design and setting

We conducted a retrospective cohort study of female subjects with FQSEC UTIs who received FQ therapy at outpatient practices within University of Pennsylvania Health System (UPHS). Our study population was the subset of an ambulatory FQSEC UTI cohort at UPHS (n = 2001). A detailed description of the UPHS cohort has been published elsewhere [8]. The study was approved by the University of Pennsylvania Institutional Review Board.

### Inclusion criteria and study definition

We enrolled female adults (age ≥ 18 years) who met the study definition for FQSEC UTIs and received any FQ antibiotic as the first antibiotic regimen for treatment of UTI within 72 h before or after obtaining an index urine culture. Exposed patients were those with high MIC FQSEC-UTIs and unexposed patients were those with low MIC-FQSEC UTIs.

Either upper or lower UTIs were eligible for the study. If an eligible patient had more than one episode of UTI during the study period, only the first episode was included. Patients who had a UTI episode within 30 days prior to the beginning of the study were also excluded. All forms of FQ antibiotic including oral form, intravenous form (outpatient antimicrobial therapy) and IV-to-PO switching were eligible. The index date was the first date of FQ therapy.

Study definitions of UTIs are shown in Table 1. For the treatment outcome, a given patient was documented as having treatment failure if at least one of the following criteria were met within 10 weeks after initiation of FQ therapy; (1) a second course of antibiotic therapy for UTI was prescribed; (2) any evidence of persistent or recurrent *E. coli* bacteriuria (At least 10^3^ cfu/mL of *E. coli* isolate).

**Table 1:** Study definition of urinary tract infections (UTIs)

To be diagnosed of UTI, an eligible subject must meet both criterion 1 and criterion 2	
Criterion 1	Having a positive urine culture ≥ 10^5^ cfu/mL, with no more than two species of microorganism
Criterion 2	At least one of the following
	ICD-9 code of signs and symptoms of UTIs
	Dipstick test positive for leukocyte esterase and/or nitrate
	Pyuria (≥ 10 white blood cells (wbc)/mm^3^ or ≥ 3 wbc/high power field of unspun urine)
	Physician diagnosis of a urinary tract infection (ICD-9 code)
	599.0 Urinary tract infection, site not specified
	590.x Infection of kidney
	595.0 Acute cystitis
	597.x Urethritis, not sexually transmitted diseases

### Microbiological test

Microbiological tests were routinely processed at the Hospital of University of Pennsylvania microbiology laboratory (HUP MicroLab). All tests were processed by the Vitek-2 system (bioMerieux Inc.), according to the performance standards for antimicrobial susceptibility testing established by Clinical and Laboratory Standards Institute (CLSI) [1]. The Vitek card used in our study provides a resulting range of seven MIC doubling dilution (≤ 0.12, 0.25, 0.5, 1, 2, 4 and ≥ 8).

An *E. coli* isolate with the levofloxacin MIC ≤ 2 mg/L was considered FQSEC. The low MIC isolates were those FQSEC with the levofloxacin MIC ≤ 0.12 mg/L while the high MIC isolates were those FQSEC isolates with the levofloxacin MIC > 0.12 but ≤ 2 mg/L.

### Data collection

Baseline characteristics were obtained via our integrated electronic clinical database called Penn Data Store (including outpatient and inpatient electronic medical records, laboratory database and billing database). Microbiological results were obtained via the HUP MicroLab laboratory information system. Chart-review was performed by the principal investigator to determine treatment outcomes.

### Statistical analysis

Categorical variables were analyzed using the Chi square or Fisher’s exact test and continuous variables were compared using the student’s t or Mann–Whitney U test, depending on the sample distribution. Mantel–Haenszel method was used for adjusting of single potential confounder. A two-tailed *p* value of < 0.05 was considered statistically significant. All calculations were performed using the STATA version 12.0 (Stata Corp, College Station TX).

## Results

During a 3-year study period (May 1, 2008–April 30, 2011), a total of 279 eligible patients were identified. However, only 275 study patients had available medical record data for review. Of these 275 patients, there were 29 patients in the high MIC-FQSEC group and 246 patients in the low MIC-FQSEC group. Median age (range) of the high MIC-FQSEC group and the low MIC-FQSEC group were 64 [18–89] years and 55 [18–99] years, respectively. Four of patients in the high MIC-FQSEC group and four patients in the low MIC-FQSEC group were treated with intravenous fluoroquinolone for a few days before switching to the oral form. Baseline characteristics between the high MIC-FQSEC and the low MIC-FQSEC groups are comparable as shown in Table 2.

**Table 2:** Baseline characteristics of patients in the low MIC group vs. the high MIC group

Variables	High MIC (N = 29)		Low MIC (N = 246)		*p* value
	N	%	N	%	
Median age [range]	64 [18–89]		55 [18–99]		0.35*
Race					
White	15	51.7	89	36.2	0.14*
Black	11	37.9	141	57.3	
Asian	1	3.5	4	1.6	
Other/unknown	2	6.9	12	4.9	
Co-morbidity					
Median Charlson index [range]	0 [0–2]		0 [0–2]		0.44*
Having at least one Charlson conditions	8	27.6	54	22.0	0.49
Acute myocardial infarction	1	3.5	4	1.6	0.43*
Congestive heart failure	3	10.3	8	3.3	0.10*
Peripheral vascular disease	0	0.0	6	2.4	0.99*
Cerebrovascular disease	2	6.9	12	4.9	0.65*
Dementia	0	0.0	3	1.2	0.99*
COPD	1	3.5	14	5.7	0.99*
Rheumatoid disease	0	0.0	1	0.4	0.99*
Peptic ulcer	0	0.0	1	0.4	0.99*
Mild liver disease	1	3.5	0	0.0	0.11*
Moderate/severe liver disease	0	0.0	0	0.0	–
Diabetes	1	3.5	3	1.2	0.36*
Hemiplegia or paraplegia	0	0.0	1	0.4	0.99*
Renal disease	2	6.9	6	2.4	0.20*
Cancer	1	3.5	12	4.9	0.99*
Metastatic cancer	0	0.0	7	2.9	0.99*
AIDS	0	0.0	0	0.0	–

* *p* value from the non-parametric test

Two patients in the high MIC-FQSEC group and two patients in the low MIC-FQSEC group experienced treatment failure (6.9 vs. 0.8%; *p* = 0.06). All four failure cases had persistent signs or symptoms of UTIs and subsequently required a second course of antibiotic therapy. Details of treatment failure are available as shown in Table 3. Of note, only one of the four had a follow-up urine culture and that given culture did not meet criteria for significant bacteriuria. Risk difference (RD) and risk ratio (RR) for treatment failure comparing high vs. low MIC groups were 0.06 [95% CI −0.03–0.15; exact-*p* = 0.06] and 8.48 [95% CI 1.24–57.97; exact-*p* = 0.06], respectively.

**Table 3:** Detail of treatment failure episodes

Findings	High MIC group		Low MIC group	
	Patient-1	Patient-2	Patient-3	Patient-4
Type of UTIs	Acute pyelonephritis	Cystitis	Cystitis	Cystitis
First antibiotic prescription	Levofloxacin 500 mg IV once daily for 4 days	Ciprofloxacin 500 mg PO twice per day for 7 days	Ciprofloxacin 500 mg PO twice per day for 5 days	Levofloxacin 500 mg PO once daily for 7 days
Date of documented treatment failure (after the index date)	Day-4	Day-9	Day-12	Day-8
Evidence of treatment failure	Persistent fever on day-4 Levofloxacin was discontinued and IV cefipime was prescribed	Dysuria persisted on day- 9 Ciprofloxacin 500 mg PO bid for 7 days was prescribed on day-9	Dysuria persisted on day-12 Levofloxacin 500 mg PO od for 5 days was prescribed on day-12	Dysuria persisted on day-8 Nitrofurantoin 100 mg PO bid for 7 days was prescribed on day-8
Repeated urine culture	No	No	Yes (on day-12) Culture: no growth	No

Baseline characteristics of patients who experienced (n = 4) and who did not experience treatment failure (n = 271) are shown in Table 4. In the bivariable analysis, treatment failure was significantly associated with race (*p* = 0.04) and underlying cerebrovascular diseases (*p* = 0.01). After adjusting for having underlying cerebrovascular disease, we found that patients with high MIC-FQSEC UTIs were approximately seven times more likely to experience treatment failure after receiving FQ therapy comparing with those with low MIC-FQSEC UTIs (RR = 7.12; 95% CI 1.20–42.10]; MH-*p* value = 0.04). Race was not found to be a significant confounding factor.

**Table 4:** Baseline characteristics of patient in the treatment failure group vs. the no treatment failure group

Variables	Failure (n = 4)		No failure (n = 271)		*p* value* %
	N	%	N	%	
Median age [range]	65.5 [45–87]		57.0 [18–99]		0.37
Race					
White	3	75.0	101	37.3	0.04
Black	0	0.0	152	56.1	
Asian	0	0.0	5	1.9	
Other/unknown	1	25.0	13	4.8	
Co-morbidity					
Median Charlson index [range]	0 [0–2]		0 [0–2]		0.19
Having at least one Charlson conditions	2	50.0	60	22.1	0.22
Acute myocardial infarction	1	25.0	4	1.5	0.07
Congestive heart failure	0	0.0	11	4.1	0.99
Peripheral vascular disease	0	0.0	6	2.2	0.99
Cerebrovascular disease	2	50.0	12	4.4	0.01
Dementia	0	0	3	1.1	0.99
COPD	0	0	15	5.5	0.99
Rheumatoid disease	0	0	1	0.4	0.99
Peptic ulcer	0	0	1	0.4	0.99
Mild liver disease	0	0	1	0.4	0.99
Moderate/severe liver disease	0	0	0	0	–
Diabetes	0	0	4	1.5	0.99
Hemiplegia or paraplegia	0	0	1	0.4	0.99
Renal disease	0	0	8	3.0	0.99
Cancer	0	0	13	4.8	0.99
Metastatic cancer	0	0	7	2.5	0.99
AIDS	0	0	0	0	–

* *p* value from the non-parametric test

## Discussion

Based on data from our study, the FQ treatment failure rate was only 0.8% in the low MIC group and 6.9% in the high MIC group. Although the failure rate was not high, the patients with high MIC-FQSEC UTIs was seven times more likely to experience treatment failure after adjusting for underlying cerebrovascular disease.

Based on this finding, FQ therapy should be carefully given to only FQSEC-UTI cases with a low risk for reduced FQ susceptibility. Use a higher dose of FQ may resolve this problem. However, the US Food and Drug Administration has recently advised to avoid FQ therapy for mild conditions including uncomplicated UTIs because of its serious side effects. FQs should be reserved for only those who do not have alternative treatment options.

We believe that our study has several strengths. First, our study definition to identify ambulatory FQSEC UTIs has shown promising discrimination ability in our pilot study (87.8% sensitivity and 85.7% specificity). For this reason, only patients with true UTIs were enrolled into our study. Although the negative impact of high MIC-FQ susceptibility on treatment response to FQ therapy has been previously documented in infections caused by Salmonella enteric serovar Typhi (*S. typhi*) [5, 6], our study was the first study exploring this issue in *E.coli* uropathogen.

Our study had several potential limitations. Since the patients with high MIC-FQSEC UTIs may be sicker than patients with low MIC-FQSEC UTIs, this may result in a higher rate of treatment failure among the high MIC group. Due to a small sample size, we may not be able to adjust for all potential confounders. Second, it is still possible that we may overlook some failure event, although we used the specifically designed criteria to detect treatment failure. Since patients who experience treatment failure may seek a second opinion from other medical providers, treatment failure could be underestimated. To address this issue, we performed chart-review to identify documented off-network visit and treatment failure. Of these 275 study patients, there was only one documented off-network visit occurred within 3 months after the index date. This off-network visit occurred in the low MIC group (0.4%, 1/275) and it was not correlated to the UTI episode. Therefore, information bias due to off-network visits should be very minimal. Another potential limitation is generalizability. This study primarily focused on female patients with non-recurrent ambulatory FQSEC-UTIs, therefore, the results of this study may not be applicable to recurrent UTIs, UTIs caused by other pathogens, other sites of infection as well as UTIs in the non-ambulatory setting. The last but very important limitation is a very low number of treatment failure (n = 4). The significant difference identified in this study may happen by chance.

## Conclusion

Based on the study results, high MIC-FQ susceptibility (or reduced FQ susceptibility) was associated with higher rates of treatment failure among female patients with ambulatory FQSEC UTIs after adjusting for underlying cerebrovascular disease. However, there were only few cases with treatment failure identified in this study. Future studies with a larger sample size are in need to confirm these findings.

Furthermore, the US Food and Drug Administration has recently advised to avoid FQ therapy for mild conditions including uncomplicated UTIs because of its serious side effects. FQs should be reserved for only those who do not have alternative treatment options.
